# An Eye Capturing Clutch – An Orbital Foreign Body

**DOI:** 10.7759/cureus.15867

**Published:** 2021-06-23

**Authors:** Deepsekhar Das, Pallavi Singh, Sujeeth Modaboyina, Mandeep S Bajaj, Sahil Agrawal

**Affiliations:** 1 Ophthalmology, All India Institute of Medical Sciences, New Delhi, New Delhi, IND; 2 Ophthalmology, Civil Hospital, Ahmedabad, Ahmedabad, IND

**Keywords:** orbital trauma, motorcycle handle, orbit fracture, orbital foreign body, large foreign body

## Abstract

Foreign bodies inside the orbital cavity are rare. They may lead to serious complications, depending on their nature, size and mechanism of injury. A 29-year-old male presented with a motorcycle handle embedded in his left orbit, with the vision unaffected. Active wound bleeding, increasing hematoma, a low haemoglobin level, signs of hypovolemic shock, ocular acuity and mobility were investigated. A computed tomography scan revealed a long bent metallic object lodged between the globe and floor of the left orbit with fracture of the medial orbital wall and ethmoidal hemosinus with an intact cribriform plate of the ethmoid. An interdepartmental collaborative effort of Oculoplasty, Oro-Maxillofacial and Neurosurgery were utilized in the removal of the foreign body. The patient recovered well after the surgery and a course of antibiotic therapy.

A single large round-tipped foreign body in orbit composed of both metal and plastic is an extremely rare incident and fortunately in our case, was relatively harmless despite its large size. The diagnosis and management of intraorbital foreign bodies must be tailored according to their type and a proper localisation by all possible means, blunt dissection, careful haemostasis aided with good lighting, and exposure helps in their atraumatic removal.

## Introduction

Intra-orbital foreign bodies (FBs) are sequelae of facial trauma with an object. The incidence of intra-orbital FB is high in military practices; however, their occurrence in civilian life is uncommon and are predominantly due to road traffic accidents. A high-velocity injury, like a gunshot or an industrial accident, usually precedes the situation [1,2].

## Case presentation

**Figure 1:**
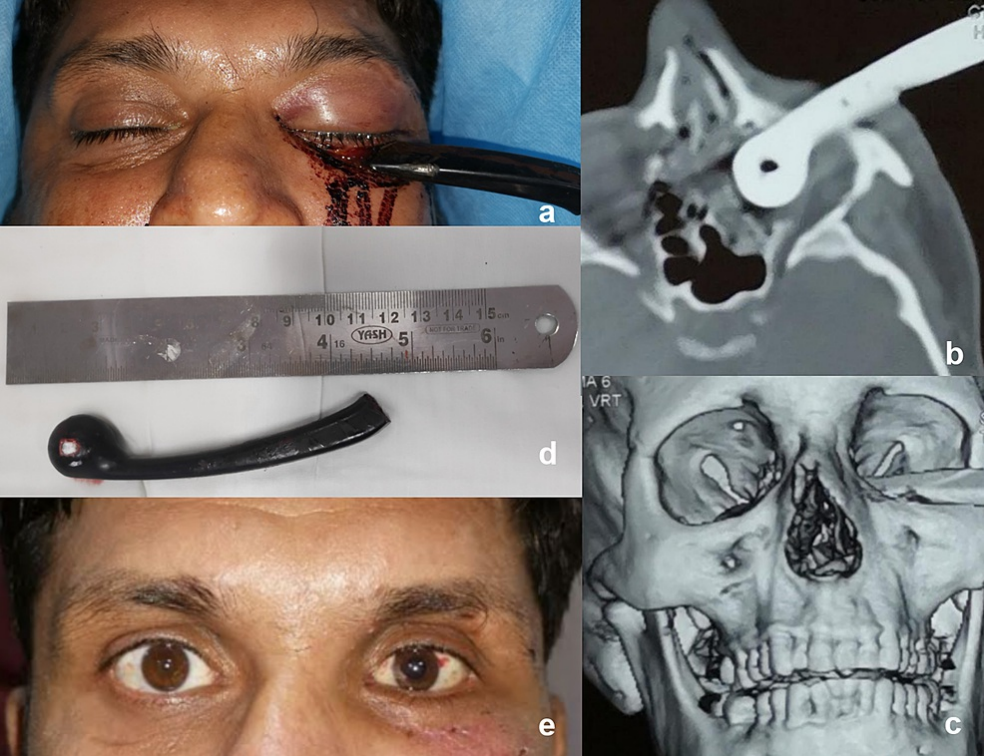
(a) Clinical photograph of the patient at presentation. (b) CT scan showing a well-defined hyperdense foreign body in the left orbit. (c) 3D reconstructed CT scan image showing a large foreign body in the left orbit. (d) Picture of the foreign body measuring 10.3cm x 2cm. (e) Clinical photograph of the patient after three weeks.

A 29-year-old male patient was brought to the emergency department with a history of road traffic accident (Figure 1, Panel a). His attendant narrated that the handle of motorcycle entered his left orbit when he suddenly lost control and the motorcycle toppled. The handle was sawed from the motorcycle to free him at the spot of the accident.

On local examination, a motorcycle handle was seen lodged in his left orbit, passing through the lower eyelid. His left upper and lower eyelids were swollen and chemosed. There was a severe limitation of extraocular muscle movements in the left eye. Visual acuity was 6/18 in the left eye with no relative apparent pupillary defect. On indirect ophthalmoscopy, posterior segment examination of both eyes was normal. There was no evidence of any injury to any other parts of the body.

A computed tomography (CT) scan of the head and orbit revealed a well-defined iso-dense structure in the left orbit lying in between the globe and the floor (Figure 1, Panels b, c). There was a medial wall fracture with bleed in the ethmoid sinus; however, there was no damage to any major vessels or the brain tissue. The cribriform plate of the ethmoid was intact. Under general anaesthesia, the wound site was examined for any hidden FBs; minor bleeders were cauterized and the wound was later irrigated with normal saline to wash remnant small FBs. A forced duction test was performed at the end, which revealed all extraocular muscles to be free.

The FB was a metallic handle measuring 10.3 cm in length and 2 cm in diameter (Figure 1, Panel d). At three-week follow-up, the patient had 6/6 visual acuity in both the eyes and no limitation of extraocular muscle movement (Figure 1, Panel e).

## Discussion

Intra-orbital FBs are infrequent situations; they have an incidence of 2.9%. The common causes for intraorbital FBs are gunshot injuries, industrial mishaps and road traffic accident as in our case [3]. They are classified based on their chemical composition into metallic, non-metallic, and organic FBs [4]. We report this case for multiple reasons. Firstly, the FB was not purely metallic and had a plastic coating on top of the metal rod; injury with such objects is limited in the literature. Secondly, the shape of the tip was rounded, whereas most of the orbital FBs reported have a sharp tip. Thirdly, even though the size of the object was exceptionally large (10.3cm x 2cm), the visual acuity even at presentation was not significantly compromised.

The clinical presentation for orbital FBs varies from being asymptomatic to having visual disturbances, pain and swelling. They may not always be as evident as in our case, a small FB may get hidden in orbit and the surrounding orbital fat can conceal the trajectory. In such cases, the patient may have persistent inflammatory signs, limitation of ocular movements and diplopia.

A thorough radiological assessment aids in pinpointing the exact site of the FB. It can help in roughly estimating the nature and size, and the amount of peri-orbital tissue inflammation. It also provides vital information regarding the integrity of the globe. The choice of imaging modality is determined by the suspected nature of the FB [2].

Plain X-ray can be utilized to locate the FBs; however, these films lack the ability to provide the exact location in relation to adjacent tissue response or surrounding damage [2]. Ultrasonography requires expertise and needs a lot of time [4]. Magnetic resonance imaging is contraindicated whenever there is a suspicion of a metallic FB, as the magnetic field can cause movement of the metallic structure [4]. Therefore, CT scan is considered to be the gold standard for diagnosing intraorbital FBs [5]. A metallic FB may have an artefact effect on a CT scan. CT findings of wooden FBs vary over time. In the acute stages, the wood can mimic air bubbles; in the subacute stages, wood may look like orbital fat and in the chronic stage, wood can imitate extraocular muscles [6,7]. In our scenario, CT scan images, both 2D and 3D reconstructed images provided extensive details regarding the dimensions of the FB as well as its extent into the orbit.

Surgical removal of the FB is indicated in situations as a large or sharp-edged FB, presence of signs of infection or inflammation, evidence of proptosis, restricted motility, presence of optic nerve compression, etc. [2]. The surgery may require a multidisciplinary approach in situations where the FB penetrates the orbital wall and enters the cranium, the adjacent sinuses, or is associated with complex facial fractures.

The literature suggests that in cases of inorganic FBs, the need for surgery depends on their location [8]. Anteriorly located FB is to be removed, whereas FBs located posteriorly without any clinical features should be left as such, as their removal may result in grave complications [9].

Metals and glass are well tolerated, and if not causing any symptoms or signs, may be left in situ [10]. All organic FBs should be removed as they may later cause complications [11]. In our case, we were able to take out the FB without causing any disruption of adjacent vital structures and had satisfactory follow-up.

## Conclusions

Large intraorbital foreign bodies may not always be associated with severe vision loss or gross ocular morbidity. A multidisciplinary approach with timely intervention can yield excellent outcomes.
